# Supplementary material for: Impact of the COVID-19 Pandemic on Adolescents’ Sexual and Reproductive Health in Low- and Middle-Income Countries
Source: Int J Environ Res Public Health. 2021 Dec 15;18(24):13221. doi: 10.3390/ijerph182413221 (PMC8701118; doi:10.3390/ijerph182413221)
Supplement: Supplementary file 1 [file ijerph-18-13221-s001.zip › ijerph-1458726-supplementary.pdf]

Details for the Databases Searched

1. **Database:** SocIndex via EBSCOhost
   Date of Search: February 19 2020

Line number	Search terms	Number of results
**S1**	((coronavirus or "corona virus" or COVID19 or COVID-19 or COVID)) OR (("SARS-Cov2" or "sars-cov-2"))	2965
**S2**	(Adolescen* or teen* or youth? or "young people" or "younger people" or "young adult*" or "young wom?n" or "young m?n" or youngster* or "school age*" or student* or "middle schooler*" or "high schooler*" or "secondary school")) AND (("Sexual health" or "sexual behavio?r*" or "sexual rights" or "sexual wellness" or "sexual wellbeing" or "sexual well-being")) OR (((adolescen* or teen*) N2 pregnan*)) OR (("Reproductive health" or "reproductive justice" or "reproductive right*" or contracepti* or condom* or "birth control" or IUD or "intrauterine device?" or "family planning" or abortion* or abstinen* or fertility or gynecolog* or obstetric* or OBGYN or menstruation or menarche or menstrual or puberty or "sexual* matur*" or maternal or maternity or prenatal or pre-natal or antenatal or postnatal or post-natal or postpartum or post-partum)) OR ((("sexual* transmi*" N4 infection*) or "sexual* transmi* disease*" or STI or STIs or STD or STDs or STBBI or STBBIs or HIV or HepC or chlamydia or gonorrh?ea or syphili*)) OR (("human papillomavirus" or HPV or "genital wart*")) OR (("sex* education" or "reproducti* education")) OR (("gender-based violence" or "dating violence" or "partner violence" or "gender-based abuse" or "dating abuse" or "partner abuse" or "consensual sex" or rape* or "safe sex" or "sex work*")) OR (((child or teen* or adolescen*) N2 sex)) OR ((sexuality or gay or lesbian* or homosexual* or transgender or intersex* or "gender expression" or "gender identity" or two-spirit* or bisexual* or heterosexual* or LGBT or LGBTQ2 or "sexual orientation" or "trans health" or "trans rights")) OR ((circumcis* or "genital mutilat*" or "genital cutting*"))) AND (("low* middle income countr*" or LMIC or LMICs or "low income countr*" or "middle income countr*")) OR ((AFGHANISTAN or afghan? or ALBANIA* or ALGERIA* or "AMERICAN SAMOA*" or ANGOLA* or ARGENTINA* or ARMENIA* or AZERBAIJAN or BANGLADESH* or BELARUS or BELIZE or BENIN or BHUTAN* or BOLIVIA* or "BOSNIA AND HERZEGOVINA" or BOTSWANA* or BRAZIL* or BULGARIA* or "BURKINA FASO" or BURUNDI or "CABO VERDE" or CAMBODIA* or CAMEROON or "CENTRAL AFRICAN REPUBLIC" or CHAD or CHINA or chinese or COLOMBIA* or COMOROS or CONGO* or "COSTA RICA*" or "COTE D'IVOIRE" or "ivory coast" or CUBA* or DJIBOUTI or DOMINICA* or "DOMINICAN REPUBLIC" or ECUADOR* or EGYPT* or "EL SALVADOR" or "EQUATORIAL GUINEA" or ERITREA* or ESWATINI or ETHIOPIA* or FIJI* or GABON or GAMBIA* or GEORGIA* or GHANA* or GRENADA* or GUATEMALA* or GUINEA	8577

	or "GUINEA-BISSAU" or GUYANA* or HAITI* or HONDURAS or INDIA* or INDONESIA* or IRAN* or IRAQ* or JAMAICA* or JORDAN* or KAZAKHSTAN* or KENYA* or KIRIBATI or KOREA* or KOSOVO* or "KYRGYZ REPUBLIC" or LAO* or LEBANON or Lebanese or LESOTHO or LIBERIA* or LIBYA* or MADAGASCAR* or MALAWI* or MALAYSIA* or MALDIVES or MALI or "MARSHALL ISLANDS" or MAURITANIA* or MEXICO or mexican or MICRONESIA or MOLDOVA* or MONGOLIA* or MONTENEGRO or MOROCCO or moroccan* or MOZAMBIQUE or MYANMAR or NAMIBIA* or NEPAL* or NICARAGUA* or NIGER or NIGERIA* or "NORTH MACEDONIA" or PAKISTAN* or "PAPUA NEW GUINEA" or PARAGUAY or PERU* or PHILIPPINES or filipino* or RUSSIA* or RWANDA* or SAMOA* or "SAO TOME AND PRINCIPE" or SENEGAL* or SERBIA* or "SIERRA LEONE" or "SOLOMON ISLANDS" or SOMALIA* or "SOUTH AFRICA*" or "SRI LANKA*" or "ST. LUCIA*" or "ST. VINCENT AND THE GRENADINES" or SUDAN* or SURINAME or SYRIA* or TAJIKISTAN* or TANZANIA* or THAILAND or thai or "TIMOR-LESTE" or TOGO or TONGA* or TUNISIA* or TURKEY or turkish or TURKMENISTAN* or TUVALU or UGANDA* or UKRAINE or ukrainian* or UZBEKISTAN* or VANUATU or VENEZUELA* or VIETNAM or Vietnamese or "WEST BANK AND GAZA" or YEMEN or ZAMBIA* or ZIMBABWE*))	
**S3**	S1 and S2	4

2. **Database**: Preliminary Medline Search via OVID
   Date of search: January, 21 2021
   Publication years: Ovid MEDLINE(R) ALL <1946 to January 19, 2021>

Line Number	Search terms	Number of Results
1	((((exp Coronavirus/ or exp Coronavirus Infections/ or (D614G or coronavirus* or corona virus* or OC43 or NL63 or 229E or HKU1 or HCoV* or ncov* or covid* or sars-cov* or sarscov* or Sars-coronavirus* or Severe Acute Respiratory Syndrome Coronavirus*).mp.) and ((20191* or 202*).dp. or 20190101:20301231.(ep).)) not (SARS or SARS-CoV or MERS or MERS-CoV or Middle East respiratory syndrome or camel* or dromedar* or equine or coronary or coronal or covidence* or covidien or influenza virus or HIV or bovine or calves or TGEV or feline or porcine or BCoV or PED or PEDV or PDCoV or FIPV or FCoV or SADS-CoV or canine or	98196

	CCov or zoonotic or avian influenza or H1N1 or H5N1 or H5N6 or IBV or murine corona*).mp.) or ((((pneumonia or covid* or coronavirus* or corona virus* or ncov* or 2019-ncov or sars*).mp. or exp pneumonia/) and Wuhan.mp.) or (2019-ncov or ncov19 or ncov-19 or 2019-novel CoV or sars-cov2 or sars-cov-2 or sarscov2 or sarscov-2 or Sars-coronavirus2 or Sars-coronavirus-2 or SARS-like coronavirus* or coronavirus-19 or covid19 or covid-19 or covid 2019 or ((novel or new or nouveau) adj2 (CoV on nCoV or covid or coronavirus* or corona virus or Pandemi*2)) or ((covid or covid19 or covid-19) and pandemic*2) or (coronavirus* and pneumonia)).mp. or COVID-19.rx,px,ox. or severe acute respiratory syndrome coronavirus 2.os.)) and 20191201:20301231.(dt).	
2	adolescent/ or young adult/ or (Adolescen* or teen* or youth? or "young people" or "younger people" or "young adult*" or "young women" or "young men" or "school age*" or student* or "middle schooler*" or "high schooler*" or "secondary school").mp.	2885549
3	Sexual Health/ or exp Sexual Behavior/	109570
4	Reproductive Rights/	959
5	("Sexual health" or "sexual behavio?r*" or "sexual rights" or "sexual wellness" or "sexual wellbeing" or "sexual well-being").mp.	97923
6	Reproductive Health/	3589
7	exp contraceptive agents/ or exp contraceptive agents, female/ or exp contraceptives, oral/ or exp contraceptives, postcoital/ or exp contraceptive agents, hormonal/ or exp contraceptive agents, male/	74417
8	contraceptive devices/ or exp contraceptive devices, female/ or exp intrauterine devices/ or exp intrauterine devices, medicated/ or exp contraceptive devices, male/	25611
9	pregnancy/ or pregnancy in adolescence/ or pregnancy, unplanned/ or pregnancy, unwanted/ or ((adolescen* or teen*) adj2 pregnan*).mp.	890963
10	exp Abortion, Induced/	40453
11	Fertility/	40515

12	("Reproductive health" or "reproductive justice" or "reproductive right*" or contracepti* or condom* or "birth control" or IUD or "intrauterine device?" or "family planning" or abortion* or abstinen* or fertility).mp.	337713
13	Sexually transmitted diseases/ or exp Sexually transmitted diseases, bacterial/ or exp Hepatitis C/ or exp HIV/ or (("sexual* transmi*" adj4 infection*) or "sexual* transmi* disease*" or STI or STIs or STD or STDs or STBBI or STBBIs or HIV or HepC or chlamydia or gonorrh?ea or syphili*).mp.	518131
14	exp sexually transmitted diseases, viral/ or exp hiv infections/	297105
15	exp Papillomavirus Vaccines/	8173
16	("human papillomavirus" or HPV).mp.	52877
17	Sex Education/	8864
18	("sex* education" or "reproducti* education").mp.	13969
19	gender-based violence/ or intimate partner violence/ or physical abuse/ or rape/ or Sex Offenses/	17924
20	("gender-based violence" or "dating violence" or "partner violence" or "gender-based abuse" or "dating abuse" or "partner abuse" or "consensual sex" or rape* or "safe sex" or "sex work*").mp.	41800
21	health services accessibility/ or right to health/	76943
22	exp Reproductive Health Services/ or "planned parenthood".mp.	40972
23	((child or teen* or adolescen*) adj2 sex).mp.	2536
24	exp sexuality/ or exp homosexuality/	42605
25	(sexuality or gay or lesbian* or homosexual* or transgender or intersex* or "gender expression" or "gender identity" or two-spirit* or bisexual* or heterosexual* or LGBT or LGBTQ2 or "sexual orientation" or "trans health" or "trans rights").mp.	94579
26	body modification, non-therapeutic/ or circumcision, female/ or circumcision, male/	6671
27	(circumcis* or "genital mutilat*" or "genital cutting*").mp.	9385
28	or/3-27	1899897
29	Developing Countries/	75877

30	("low* middle income countr*" or LMIC or LMICs).mp.	7643
31	(AFGHANISTAN or ALBANIA or ALGERIA or "AMERICAN SAMOA" or ANGOLA or ARGENTINA or ARMENIA or AZERBAIJAN or BANGLADESH or BELARUS or BELIZE or BENIN or BHUTAN or BOLIVIA or "BOSNIA AND HERZEGOVINA" or BOTSWANA or BRAZIL or BULGARIA or "BURKINA FASO" or BURUNDI or "CABO VERDE" or CAMBODIA or CAMEROON or "CENTRAL AFRICAN REPUBLIC" or CHAD or CHINA or COLOMBIA or COMOROS or CONGO or "COSTA RICA" or "COTE D'IVOIRE" or CUBA or DJIBOUTI or DOMINICA or "DOMINICAN REPUBLIC" or ECUADOR or EGYPT or "EL SALVADOR" or "EQUATORIAL GUINEA" or ERITREA or ESWATINI or ETHIOPIA or FIJI or GABON or GAMBIA or GEORGIA or GHANA or GRENADA or GUATEMALA or GUINEA or "GUINEA-BISSAU" or GUYANA or HAITI or HONDURAS or INDIA or INDONESIA or IRAN or IRAQ or JAMAICA or JORDAN or KAZAKHSTAN or KENYA or KIRIBATI or KOREA or KOSOVO or "KYRGYZ REPUBLIC" or LAO or LEBANON or LESOTHO or LIBERIA or LIBYA or MADAGASCAR or MALAWI or MALAYSIA or MALDIVES or MALI or "MARSHALL ISLANDS" or MAURITANIA or MEXICO or MICRONESIA or MOLDOVA or MONGOLIA or MONTENEGRO or MOROCCO or MOZAMBIQUE or MYANMAR or NAMIBIA or NEPAL or NICARAGUA or NIGER or NIGERIA or "NORTH MACEDONIA" or PAKISTAN or "PAPUA NEW GUINEA" or PARAGUAY or PERU or PHILIPPINES or RUSSIA or RWANDA or SAMOA or "SAO TOME AND PRINCIPE" or SENEGAL or SERBIA or "SIERRA LEONE" or "SOLOMON ISLANDS" or SOMALIA or "SOUTH AFRICA" or "SRI LANKA" or "ST. LUCIA" or "ST. VINCENT AND THE	1633635

	GRENADINES" or SUDAN or SURINAME or SYRIA or TAJIKISTAN or TANZANIA or THAILAND or "TIMOR-LESTE" or TOGO or TONGA or TUNISIA or TURKEY or TURKMENISTAN or TUVALU or UGANDA or UKRAINE or UZBEKISTAN or VANUATU or VENEZUELA or VIETNAM or "WEST BANK AND GAZA" or YEMEN or ZAMBIA or ZIMBABWE).mp.	
32	29 or 30 or 31	1671969
33	1 and 2 and 28 and 32	91

3. **Database**: Medline via OVID (1946 - Present)
   Date of search: Feb 19 2021
   Ovid MEDLINE(R) ALL <1946 to February 18, 2021>

Line Number	Search terms	Number of results
1	((((exp Coronavirus/ or exp Coronavirus Infections/ or (D614G or coronavirus* or corona virus* or OC43 or NL63 or 229E or HKU1 or HCoV* or ncov* or covid* or sars-cov* or sarscov* or Sars-coronavirus* or Severe Acute Respiratory Syndrome Coronavirus*).mp.) and ((20191* or 202*).dp. or 20190101:20301231.(ep).)) not (SARS or SARS-CoV or MERS or MERS-CoV or Middle East respiratory syndrome or camel* or dromedar* or equine or coronary or coronal or covidence* or covidien or influenza virus or HIV or bovine or calves or TGEV or feline or porcine or BCoV or PED or PEDV or PDCoV or FIPV or FCoV or SADS-CoV or canine or CCov or zoonotic or avian influenza or H1N1 or H5N1 or H5N6 or IBV or murine corona*).mp.) or ((((pneumonia or covid* or coronavirus* or corona virus* or ncov* or 2019-ncov or sars*).mp. or exp pneumonia/) and Wuhan.mp.) or (2019-ncov or ncov19 or ncov-19 or 2019-novel CoV or sars-cov2 or sars-cov-2 or sarscov2 or sarscov-2 or Sars-coronavirus2 or Sars-coronavirus-2 or SARS-like coronavirus* or coronavirus-19 or covid19 or covid-19 or covid 2019 or ((novel or new or nouveau) adj2 (CoV on nCoV or covid or coronavirus* or corona virus or Pandemi*2)) or ((covid or covid19 or covid-19) and pandemic*2) or (coronavirus* and pneumonia)).mp. or COVID-19.rx,px,ox. or severe acute respiratory	107879

	syndrome coronavirus 2.os.)) and 20191201:20301231.(dt).	
2	adolescent/ or young adult/ or (Adolescen* or teen* or youth? or "young people" or "younger people" or "young adult*" or "young wom?n" or "young m?n" or youngster* or "school age*" or student* or "middle schooler*" or "high schooler*" or "secondary school").mp.	2910465
3	Sexual Health/ or exp Sexual Behavior/	109994
4	Reproductive Rights/ or exp menstrual cycle/ or exp puberty/ or exp reproduction/ or reproductive behavior/ or exp contraception behavior/ or exp sexual development/ or gynecology/ or obstetrics/	1257613
5	("Sexual health" or "sexual behavio?r*" or "sexual rights" or "sexual wellness" or "sexual wellbeing" or "sexual well-being").mp.	98422
6	Reproductive Health/	3635
7	exp contraceptive agents/ or exp contraceptive agents, female/ or exp contraceptives, oral/ or exp contraceptives, postcoital/ or exp contraceptive agents, hormonal/ or exp contraceptive agents, male/	74501
8	contraceptive devices/ or exp contraceptive devices, female/ or exp intrauterine devices/ or exp intrauterine devices, medicated/ or exp contraceptive devices, male/	25658
9	pregnancy/ or pregnancy in adolescence/ or pregnancy, unplanned/ or pregnancy, unwanted/ or ((adolescen* or teen*) adj2 pregnan*).mp.	893878
10	exp Abortion, Induced/	40520
11	Fertility/	40698
12	("Reproductive health" or "reproductive justice" or "reproductive right*" or contracepti* or condom* or "birth control" or IUD or "intrauterine device?" or "family planning" or abortion* or abstinen* or fertility or gynecolog* or obstetric* or OBGYN or menstruation or menarche or menstrual or puberty or "sexual* matur*" or maternal or maternity or prenatal or pre-natal or antenatal or postnatal or post-natal or postpartum or post-partum).mp.	1151371
13	Sexually transmitted diseases/ or exp Sexually transmitted diseases, bacterial/ or exp Hepatitis C/ or exp HIV/ or (("sexual* transmi*" adj4 infection*) or "sexual* transmi* disease*" or STI or STIs or STD or STDs or STBBI or STBBIs or HIV or HepC or chlamydia or gonorrh?ea or syphili*).mp.	520016

14	exp sexually transmitted diseases, viral/ or exp hiv infections/	298091
15	exp Papillomavirus Vaccines/	8219
16	("human papillomavirus" or HPV).mp.	53211
17	Sex Education/	8874
18	("sex* education" or "reproducti* education").mp.	14048
19	gender-based violence/ or intimate partner violence/ or physical abuse/ or rape/ or Sex Offenses/	18046
20	("gender-based violence" or "dating violence" or "partner violence" or "gender-based abuse" or "dating abuse" or "partner abuse" or "consensual sex" or rape* or "safe sex" or "sex work*").mp.	42066
21	health services accessibility/ or right to health/	77472
22	exp Reproductive Health Services/ or "planned parenthood".mp.	41126
23	((child or teen* or adolescen*) adj2 sex).mp.	2562
24	exp sexuality/ or exp homosexuality/	42785
25	(sexuality or gay or lesbian* or homosexual* or transgender or intersex* or "gender expression" or "gender identity" or two-spirit* or bisexual* or heterosexual* or LGBT or LGBTQ2 or "sexual orientation" or "trans health" or "trans rights").mp.	95135
26	body modification, non-therapeutic/ or circumcision, female/ or circumcision, male/	6689
27	(circumcis* or "genital mutilat*" or "genital cutting*").mp.	9418
28	or/3-27	2535211
29	Developing Countries/	76120
30	("low* middle income countr*" or LMIC or LMICs or "low income countr*" or "middle income countr*").mp.	28968
31	(AFGHANISTAN or afghan? or ALBANIA* or ALGERIA* or "AMERICAN SAMOA*" or ANGOLA* or ARGENTINA* or ARMENIA* or AZERBAIJAN or BANGLADESH* or BELARUS or BELIZE or BENIN or BHUTAN* or BOLIVIA* or "BOSNIA AND HERZEGOVINA" or BOTSWANA* or BRAZIL* or BULGARIA* or "BURKINA FASO" or BURUNDI or "CABO VERDE" or CAMBODIA* or CAMEROON or "CENTRAL AFRICAN REPUBLIC" or CHAD or CHINA or chinese or COLOMBIA* or COMOROS or CONGO* or "COSTA RICA*" or "COTE D'IVOIRE" or "ivory coast" or CUBA* or DJIBOUTI or DOMINICA* or "DOMINICAN REPUBLIC" or ECUADOR* or EGYPT* or "EL	2029292

	SALVADOR" or "EQUATORIAL GUINEA" or ERITREA* or ESWATINI or ETHIOPIA* or FIJI* or GABON or GAMBIA* or GEORGIA* or GHANA* or GRENADA* or GUATEMALA* or GUINEA or "GUINEA-BISSAU" or GUYANA* or HAITI* or HONDURAS or INDIA* or INDONESIA* or IRAN* or IRAQ* or JAMAICA* or JORDAN* or KAZAKHSTAN* or KENYA* or KIRIBATI or KOREA* or KOSOVO* or "KYRGYZ REPUBLIC" or LAO* or LEBANON or Lebanese or LESOTHO or LIBERIA* or LIBYA* or MADAGASCAR* or MALAWI* or MALAYSIA* or MALDIVES or MALI or "MARSHALL ISLANDS" or MAURITANIA* or MEXICO or mexican or MICRONESIA or MOLDOVA* or MONGOLIA* or MONTENEGRO or MOROCCO or moroccan* or MOZAMBIQUE or MYANMAR or NAMIBIA* or NEPAL* or NICARAGUA* or NIGER or NIGERIA* or "NORTH MACEDONIA" or PAKISTAN* or "PAPUA NEW GUINEA" or PARAGUAY or PERU* or PHILIPPINES or filipino* or RUSSIA* or RWANDA* or SAMOA* or "SAO TOME AND PRINCIPE" or SENEGAL* or SERBIA* or "SIERRA LEONE" or "SOLOMON ISLANDS" or SOMALIA* or "SOUTH AFRICA*" or "SRI LANKA*" or "ST. LUCIA*" or "ST. VINCENT AND THE GRENADINES" or SUDAN* or SURINAME or SYRIA* or TAJIKISTAN* or TANZANIA* or THAILAND or thai or "TIMOR-LESTE" or TOGO or TONGA* or TUNISIA* or TURKEY or turkish or TURKMENISTAN* or TUVALU or UGANDA* or UKRAINE or ukrainian* or UZBEKISTAN* or VANUATU or VENEZUELA* or VIETNAM or Vietnamese or "WEST BANK AND GAZA" or YEMEN or ZAMBIA* or ZIMBABWE*).mp.	
32	29 or 30 or 31	2073642
33	1 and 2 and 28 and 32	121
34	(intervention* or approach* or strateg* or program* or service* or plan* or policy or policies or scheme* or access* or procedure*).ti,ab.	6766485
35	33 and 34	64

4. **Database**: CINAHL via EBSCOhost (1936 - Present)
   Date of search: Feb 19 2021

Line Number	Search Terms	Search Results
S1	((MH "Coronavirus+") or coronavirus* or covid) AND (wuhan or beijing or shanghai)) OR (("novel coronavirus*" AND ((MH "China") or China)) OR TI coronavirus* OR (((MH pneumonia) or pneumonia) AND Wuhan) OR ((D614G or "Covid-19" or Covid19 or "2019-nCoV" or "SARS-CoV-2" or (MH Coronavirus Infections))))) AND ((MH "Coronavirus+") or coronavirus* or covid) AND (wuhan or beijing or shanghai)) OR (("novel coronavirus*" AND ((MH "China") or China)) OR TI coronavirus* OR (((MH pneumonia) or pneumonia) AND Wuhan) OR ((D614G or "Covid-19" or Covid19 or "2019-nCoV" or "SARS-CoV-2" or (MH Coronavirus Infections))))) AND DT 20191201-20300101)	40705
S2	(Adolescen* or teen* or youth? or "young people" or "younger people" or "young adult*" or "young wom?n" or "young m?n" or youngster* or "school age*" or student* or "middle schooler*" or "high schooler*" or "secondary school")	923838
S3	("Sexual health" or "sexual behavio?r*" or "sexual rights" or "sexual wellness" or "sexual wellbeing" or "sexual well-being")) OR (((adolescen* or teen*) N2 pregnan*)) OR (("Reproductive health" or "reproductive justice" or "reproductive right*" or contracepti* or condom* or "birth control" or IUD or "intrauterine device?" or "family planning" or abortion* or abstinen* or fertility or gynecolog* or obstetric* or OBGYN or menstruation or menarche or menstrual or puberty or "sexual* matur*" or maternal or maternity or prenatal or pre-natal or antenatal or postnatal or post-natal or postpartum or post-partum)) OR ((("sexual* transmi*" N4 infection*) or "sexual* transmi* disease*" or STI or STIs or STD or STDs or STBBI or STBBIs or HIV or HepC or chlamydia or gonorrh?ea or syphili*)) OR (("human papillomavirus" or HPV or "genital wart*")) OR (("sex* education" or "reproducti* education")) OR (("gender-based violence" or "dating violence" or "partner violence" or "gender-based	584331

	abuse" or "dating abuse" or "partner abuse" or "consensual sex" or rape* or "safe sex" or "sex work*")) OR (((child or teen* or adolescen*) N2 sex)) OR ((sexuality or gay or lesbian* or homosexual* or transgender or intersex* or "gender expression" or "gender identity" or two-spirit* or bisexual* or heterosexual* or LGBT or LGBTQ2 or "sexual orientation" or "trans health" or "trans rights")) OR ((circumcis* or "genital mutilat*" or "genital cutting*"))	
S4	("low* middle income countr*" or LMIC or LMICs or "low income countr*" or "middle income countr*")) OR ((AFGHANISTAN or afghan? or ALBANIA* or ALGERIA* or "AMERICAN SAMOA*" or ANGOLA* or ARGENTINA* or ARMENIA* or AZERBAIJAN or BANGLADESH* or BELARUS or BELIZE or BENIN or BHUTAN* or BOLIVIA* or "BOSNIA AND HERZEGOVINA" or BOTSWANA* or BRAZIL* or BULGARIA* or "BURKINA FASO" or BURUNDI or "CABO VERDE" or CAMBODIA* or CAMEROON or "CENTRAL AFRICAN REPUBLIC" or CHAD or CHINA or chinese or COLOMBIA* or COMOROS or CONGO* or "COSTA RICA*" or "COTE D'IVOIRE" or "ivory coast" or CUBA* or DJIBOUTI or DOMINICA* or "DOMINICAN REPUBLIC" or ECUADOR* or EGYPT* or "EL SALVADOR" or "EQUATORIAL GUINEA" or ERITREA* or ESWATINI or ETHIOPIA* or FIJI* or GABON or GAMBIA* or GEORGIA* or GHANA* or GRENADA* or GUATEMALA* or GUINEA or "GUINEA-BISSAU" or GUYANA* or HAITI* or HONDURAS or INDIA* or INDONESIA* or IRAN* or IRAQ* or JAMAICA* or JORDAN* or KAZAKHSTAN* or KENYA* or KIRIBATI or KOREA* or KOSOVO* or "KYRGYZ REPUBLIC" or LAO* or LEBANON or Lebanese or LESOTHO or LIBERIA* or LIBYA* or MADAGASCAR* or MALAWI* or MALAYSIA* or MALDIVES or MALI or "MARSHALL ISLANDS" or MAURITANIA* or MEXICO or mexican or MICRONESIA or MOLDOVA* or MONGOLIA* or	555881

	MONTENEGRO or MOROCCO or moroccan* or MOZAMBIQUE or MYANMAR or NAMIBIA* or NEPAL* or NICARAGUA* or NIGER or NIGERIA* or "NORTH MACEDONIA" or PAKISTAN* or "PAPUA NEW GUINEA" or PARAGUAY or PERU* or PHILIPPINES or filipino* or RUSSIA* or RWANDA* or SAMOA* or "SAO TOME AND PRINCIPE" or SENEGAL* or SERBIA* or "SIERRA LEONE" or "SOLOMON ISLANDS" or SOMALIA* or "SOUTH AFRICA*" or "SRI LANKA*" or "ST. LUCIA*" or "ST. VINCENT AND THE GRENADINES" or SUDAN* or SURINAME or SYRIA* or TAJIKISTAN* or TANZANIA* or THAILAND or thai or "TIMOR-LESTE" or TOGO or TONGA* or TUNISIA* or TURKEY or turkish or TURKMENISTAN* or TUVALU or UGANDA* or UKRAINE or ukrainian* or UZBEKISTAN* or VANUATU or VENEZUELA* or VIETNAM or Vietnamese or "WEST BANK AND GAZA" or YEMEN or ZAMBIA* or ZIMBABWE*))	
S5	TI ((intervention* or approach* or strateg* or program* or service* or plan* or policy or policies or scheme* or access* or procedure*)) OR AB ((intervention* or approach* or strateg* or program* or service* or plan* or policy or policies or scheme* or access* or procedure*))	1826667
S6	S1 AND S2 AND S3 AND S4 AND S5 (lmit to scholarly (peer reviewed) journals)	29

5. **Database**: Scopus via Elsevier (1976 - Present)
Date of search: Feb 19 2021
Search terms: (((TITLE-ABS-KEY ((coronavirus* OR "corona virus*" OR oc43 OR nl63 OR 229e OR hku1 OR hcov* OR ncov* OR covid* OR "sars-cov*" OR sarscov* OR "Sars-coronavirus*" OR "Severe Acute Respiratory Syndrome Coronavirus*" OR d614g))) AND NOT ((TITLE-ABS-KEY ((sars OR sars-cov OR mers OR mers-cov OR "Middle East respiratory syndrome or camel*" OR dromedar* OR equine OR coronary OR coronal OR covidence* OR covidien OR influenza AND virus OR hiv OR bovine OR calves OR tgev OR feline OR porcine OR bcov))) OR (TITLE-ABS-KEY ((ped OR pedv OR pdcov OR fipv OR fcov OR sads-cov OR canine OR ccov OR zoonotic OR "avian influenza" OR h1n1 OR h5n1 OR h5n6 OR ibv OR murine AND corona*))))) OR (TITLE-ABS-KEY ((pneumonia OR covid* OR coronavirus* OR corona AND virus* OR ncov* OR 2019-ncov OR sars*) AND wuhan) OR ((2019-ncov OR ncov19 OR ncov-19 OR 2019-novel AND cov

OR sars-cov2 OR sars-cov-2 OR sarscov2 OR sarscov-2 OR sars-coronavirus2 OR sars-coronavirus-2 OR "SARS-like coronavirus*" OR coronavirus-19 OR covid19 OR covid-19 OR "covid 2019" OR ((covid OR covid19 OR covid-19) AND pandemic*2) OR (coronavirus* AND pneumonia)))) OR (TITLE ((novel OR new OR nouveau) AND (cov OR ncov OR covid OR coronavirus* OR corona AND virus OR pandemi*))) OR (ABS ((novel OR new OR nouveau) AND (cov OR ncov OR covid OR coronavirus* OR corona AND virus OR pandemi*))) OR (KEY ((novel OR new OR nouveau) AND (cov OR ncov OR covid OR coronavirus* OR corona AND virus OR pandemi*)))) AND (TITLE-ABS-KEY ((adolescen* OR teen* OR youth? OR "young people" OR "younger people" OR "young adult*" OR "young wom?n" OR "young m?n" OR youngster* OR "school age*" OR student* OR "middle schooler*" OR "high schooler*" OR "secondary school"))) AND (TITLE-ABS-KEY ("Sexual health" OR "sexual behavio?r*" OR "sexual rights" OR "sexual wellness" OR "sexual wellbeing" OR "sexual well-being" OR ((adolescen* OR teen*) W/2 pregnan*) OR "Reproductive health" OR "reproductive justice" OR "reproductive right*" OR contracepti* OR condom* OR "birth control" OR iud OR "intrauterine device?" OR "family planning" OR abortion* OR abstinen* OR fertility OR gynecolog* OR obstetric* OR obgyn OR menstruation OR menarche OR menstrual OR puberty OR "sexual* matur*" OR maternal OR maternity OR prenatal OR pre-natal OR antenatal OR postnatal OR post-natal OR postpartum OR post-partum OR ("sexual* transmi*" W/4 infection*) OR "sexual* transmi* disease*" OR sti OR stis OR std OR stds OR stbbi OR stbbis OR hiv OR hepc OR chlamydia OR gonorrh?ea OR syphili* OR "human papillomavirus" OR hpv OR "genital wart*" OR "sex* education" OR "reproducti* education" OR "gender-based violence" OR "dating violence" OR "partner violence" OR "gender-based abuse" OR "dating abuse" OR "partner abuse" OR "consensual sex" OR rape* OR "safe sex" OR "sex work*" OR ((child OR teen* OR adolescen*) W/2 sex) OR sexuality OR gay OR lesbian* OR homosexual* OR transgender OR intersex* OR "gender expression" OR "gender identity" OR two-spirit* OR bisexual* OR heterosexual* OR lgbt OR lgbtq2 OR "sexual orientation" OR "trans health" OR "trans rights" OR circumcis* OR "genital mutilat*" OR "genital cutting*")) AND (TITLE-ABS-KEY ("low* middle income countr*" OR lmic OR lmics OR "low income countr*" OR "middle income countr*" OR afghanistan OR afghan? OR albania* OR algeria* OR "AMERICAN SAMOA*" OR angola* OR argentina* OR armenia* OR azerbaijan OR bangladesh* OR belarus OR belize OR benin OR bhutan* OR bolivia* OR "BOSNIA AND HERZEGOVINA" OR botswana* OR brazil* OR bulgaria* OR "BURKINA FASO" OR burundi OR "CABO VERDE" OR cambodia* OR cameroon OR "CENTRAL AFRICAN REPUBLIC" OR chad OR china OR chinese OR colombia* OR comoros OR congo* OR "COSTA RICA*" OR "COTE D'IVOIRE" OR "ivory coast" OR cuba* OR djibouti OR dominica* OR "DOMINICAN REPUBLIC" OR ecuador* OR egypt* OR "EL SALVADOR" OR "EQUATORIAL GUINEA" OR eritrea* OR eswatini OR ethiopia* OR fiji* OR gabon OR gambia* OR georgia* OR ghana* OR grenada* OR guatemala* OR guinea OR "GUINEA-BISSAU" OR guyana* OR haiti* OR honduras

OR india* OR indonesia* OR iran* OR iraq* OR jamaica* OR jordan* OR kazakhstan* OR kenya* OR kiribati OR korea* OR kosovo* OR "KYRGYZ REPUBLIC" OR lao* OR lebanon OR lebanese OR lesotho OR liberia* OR libya* OR madagascar* OR malawi* OR malaysia* OR maldives OR mali OR "MARSHALL ISLANDS" OR mauritania* OR mexico OR mexican OR micronesia OR moldova* OR mongolia* OR montenegro OR morocco OR moroccan* OR mozambique OR myanmar OR namibia* OR nepal* OR nicaragua* OR niger OR nigeria* OR "NORTH MACEDONIA" OR pakistan* OR "PAPUA NEW GUINEA" OR paraguay OR peru* OR philippines OR filipino* OR russia* OR rwanda* OR samoa* OR "SAO TOME AND PRINCIPE" OR senegal* OR serbia* OR "SIERRA LEONE" OR "SOLOMON ISLANDS" OR somalia* OR "SOUTH AFRICA*" OR "SRI LANKA*" OR "ST. LUCIA*" OR "ST. VINCENT AND THE GRENADINES" OR sudan* OR suriname OR syria* OR tajikistan* OR tanzania* OR thailand OR thai OR "TIMOR-LESTE" OR togo OR tonga* OR tunisia* OR turkey OR turkish OR turkmenistan* OR tuvalu OR uganda* OR ukraine OR ukrainian* OR uzbekistan* OR vanuatu OR venezuela* OR vietnam OR vietnamese OR "WEST BANK AND GAZA" OR yemen OR zambia* OR zimbabwe*)) AND (TITLE-ABS (intervention* OR approach* OR strateg* OR program* OR service* OR plan* OR policy OR policies OR scheme* OR access* OR procedure*)) AND (LIMIT-TO (PUBYEAR , 2021) OR LIMIT-TO (PUBYEAR , 2020) OR LIMIT-TO (PUBYEAR , 2019)) AND (LIMIT-TO (DOCTYPE , "ar") OR LIMIT-TO (DOCTYPE , "re")) View less
Number of Results: 66

6. **Database**: Web of Science (Core Collection) via Clarivate
   Date of search: February 19 2021
   Search terms: TOPIC: ((coronavirus or "corona virus" or COVID19 or COVID-19 or COVID OR "SARS-Cov2" or "sars-cov-2")) AND TOPIC: ((Adolescen* or teen* or youth? or "young people" or "younger people" or "young adult*" or "young wom?n" or "young m?n" or youngster* or "school age*" or student* or "middle schooler*" or "high schooler*" or "secondary school")) AND TOPIC: (("Sexual health" or "sexual behavio?r*" or "sexual rights" or "sexual wellness" or "sexual wellbeing" or "sexual well-being" OR ((adolescen* or teen*) NEAR/2 pregnan*) OR "Reproductive health" or "reproductive justice" or "reproductive right*" or contracepti* or condom* or "birth control" or IUD or "intrauterine device?" or "family planning" or abortion* or abstinen* or fertility or gynecolog* or obstetric* or OBGYN or menstruation or menarche or menstrual or puberty or "sexual* matur*" or maternal or maternity or prenatal or pre-natal or antenatal or postnatal or post-natal or postpartum or post-partum OR ("sexual* transmi*" NEAR/4 infection*) or "sexual* transmi* disease*" or STI or STIs or STD or STDs or STBBI or STBBIs or HIV or HepC or chlamydia or gonorrh?ea or syphili*OR "human papillomavirus" or HPV or "genital wart*" OR "sex* education" or "reproducti* education" OR "gender-based violence" or "dating violence" or "partner violence" or "gender-based abuse" or "dating abuse" or "partner abuse" or "consensual sex" or rape* or "safe sex" or "sex work*" OR ((child or teen* or adolescen*) NEAR/2 sex) OR sexuality or gay or lesbian* or homosexual* or transgender or intersex* or "gender expression" or "gender identity" or two-spirit* or bisexual* or heterosexual* or

LGBT or LGBTQ2 or "sexual orientation" or "trans health" or "trans rights" OR circumcis* or "genital mutilat*" or "genital cutting*")) AND TOPIC: (("low* middle income countr*" or LMIC or LMICs or "low income countr*" or "middle income countr*" OR AFGHANISTAN or afghan? or ALBANIA* or ALGERIA* or "AMERICAN SAMOA*" or ANGOLA* or ARGENTINA* or ARMENIA* or AZERBAIJAN or BANGLADESH* or BELARUS or BELIZE or BENIN or BHUTAN* or BOLIVIA* or "BOSNIA AND HERZEGOVINA" or BOTSWANA* or BRAZIL* or BULGARIA* or "BURKINA FASO" or BURUNDI or "CABO VERDE" or CAMBODIA* or CAMEROON or "CENTRAL AFRICAN REPUBLIC" or CHAD or CHINA or chinese or COLOMBIA* or COMOROS or CONGO* or "COSTA RICA*" or "COTE D'IVOIRE" or "ivory coast" or CUBA* or DJIBOUTI or DOMINICA* or "DOMINICAN REPUBLIC" or ECUADOR* or EGYPT* or "EL SALVADOR" or "EQUATORIAL GUINEA" or ERITREA* or ESWATINI or ETHIOPIA* or FIJI* or GABON or GAMBIA* or GEORGIA* or GHANA* or GRENADA* or GUATEMALA* or GUINEA or "GUINEA-BISSAU" or GUYANA* or HAITI* or HONDURAS or INDIA* or INDONESIA* or IRAN* or IRAQ* or JAMAICA* or JORDAN* or KAZAKHSTAN* or KENYA* or KIRIBATI or KOREA* or KOSOVO* or "KYRGYZ REPUBLIC" or LAO* or LEBANON or Lebanese or LESOTHO or LIBERIA* or LIBYA* or MADAGASCAR* or MALAWI* or MALAYSIA* or MALDIVES or MALI or "MARSHALL ISLANDS" or MAURITANIA* or MEXICO or mexican or MICRONESIA or MOLDOVA* or MONGOLIA* or MONTENEGRO or MOROCCO or moroccan* or MOZAMBIQUE or MYANMAR or NAMIBIA* or NEPAL* or NICARAGUA* or NIGER or NIGERIA* or "NORTH MACEDONIA" or PAKISTAN* or "PAPUA NEW GUINEA" or PARAGUAY or PERU* or PHILIPPINES or filipino* or RUSSIA* or RWANDA* or SAMOA* or "SAO TOME AND PRINCIPE" or SENEGAL* or SERBIA* or "SIERRA LEONE" or "SOLOMON ISLANDS" or SOMALIA* or "SOUTH AFRICA*" or "SRI LANKA*" or "ST. LUCIA*" or "ST. VINCENT AND THE GRENADINES" or SUDAN* or SURINAME or SYRIA* or TAJIKISTAN* or TANZANIA* or THAILAND or thai or "TIMOR-LESTE" or TOGO or TONGA* or TUNISIA* or TURKEY or turkish or TURKMENISTAN* or TUVALU or UGANDA* or UKRAINE or ukrainian* or UZBEKISTAN* or VANUATU or VENEZUELA* or VIETNAM or Vietnamese or "WEST BANK AND GAZA" or YEMEN or ZAMBIA* or ZIMBABWE*)

Number of Results: 21

7. **Database**: EMBASE via OVID (1974 - Feb 18 2021)
   Date of search: Feb 19 2021
   1. (((exp Coronavirus/ or exp Coronavirus Infections/ or (coronavirus* or corona virus* or OC43 or NL63 or 229E or HKU1 or HCoV* or ncov* or covid* or sars-cov* or sarscov* or Sars-coronavirus* or Severe Acute Respiratory Syndrome Coronavirus* or D614G).mp.) not (SARS or SARS-CoV or MERS or MERS-CoV or Middle East respiratory syndrome or camel* or dromedar* or equine or coronary or coronal or covidence* or covidien or influenza virus or HIV or bovine or calves or TGEV or feline or porcine or BCoV or PED or PEDV or PDCoV or FIPV or FCoV or SADS-CoV or canine or CCov or zoonotic or avian influenza or H1N1 or H5N1 or H5N6 or IBV or

murine corona*).mp.) or (((pneumonia or covid* or coronavirus* or corona virus* or ncov* or 2019-ncov or sars*).mp. or exp pneumonia/) and Wuhan.mp.) or (coronavirus disease 2019 or 2019-ncov or ncov19 or ncov-19 or 2019-novel CoV or severe acute respiratory syndrome coronavirus 2 or sars-cov2 or sars-cov-2 or sarscov2 or sarscov-2 or Sars-coronavirus2 or Sars-coronavirus-2 or SARS-like coronavirus* or coronavirus-19 or covid19 or covid-19 or covid 2019 or ((novel or new or nouveau) adj2 (CoV or nCoV or covid or coronavirus* or corona virus or Pandemi*2)) or ((covid or covid19 or covid-19) and pandemic*2) or (coronavirus* and pneumonia)).mp. or (coronavirus disease 2019 or severe acute respiratory syndrome coronavirus 2).sh,dj.) and 20191201:20301231.(dc).

2. juvenile/ or adolescent/ or adolescence/
3. young adult/
4. adolescent behavior/ or adolescent sexual behavior/ or adolescent parent/
5. (Adolescen* or teen* or youth? or "young people" or "younger people" or "young adult*" or "young wom?n" or "young m?n" or youngster* or "school age*" or student* or "middle schooler*" or "high schooler*" or "secondary school").mp.
6. 2 or 3 or 4 or 5
7. exp sexual behavior/ or exp casual sex/ or exp contraceptive behavior/ or exp polygamy/ or exp premarital sex/ or exp safe sex/ or exp sexual practice/ or exp sexual violence/ or exp unsafe sex/
8. sexual health/
9. reproductive health/
10. reproductive rights/
11. reproduction/ or exp childbirth/ or exp conception/ or exp prenatal development/ or exp puerperium/ or reproductive behavior/
12. female genital system function/ or female fertility/ or menstrual cycle/ or exp ovary function/ or exp uterus function/ or vaginal lubrication/
13. exp puberty/
14. sexual development/ or sexual maturation/ or sexual maturity/
15. medicine/ or gynecology/ or exp obstetrics/
16. ("Sexual health" or "sexual behavio?r*" or "sexual rights" or "sexual wellness" or "sexual wellbeing" or "sexual well-being").mp.
17. exp contraceptive agent/ or exp hormonal contraceptive agent/ or exp injectable contraceptive agent/ or exp male contraceptive agent/ or exp oral contraceptive agent/ or exp postcoitus contraceptive agent/ or exp spermicidal agent/
18. exp contraceptive device/ or exp female contraceptive device/ or exp male contraceptive device/
19. exp birth control implant/ or exp intrauterine contraceptive device/ or exp tubal occlusion device/ or exp vagina pessary/
20. pregnancy/ or adolescent pregnancy/ or unplanned pregnancy/ or unwanted pregnancy/
21. ((adolescen* or teen*) adj2 pregnan*).mp.
22. exp abortion/
23. exp fertility/

24. ("Reproductive health" or "reproductive justice" or "reproductive right*" or contracepti* or condom* or "birth control" or IUD or "intrauterine device?" or "family planning" or abortion* or abstinen* or fertility or gynecolog* or obstetric* or OBGYN or menstruation or menarche or menstrual or puberty or "sexual* matur*" or maternal or maternity or prenatal or pre-natal or antenatal or postnatal or post-natal or postpartum or post-partum).mp.
25. exp sexually transmitted disease/ or exp gonorrhea/ or exp syphilis/
26. exp hepatitis C/
27. exp human immunodeficiency virus/ or exp human immunodeficiency virus 1/ or exp human immunodeficiency virus 2/
28. exp Chlamydia/
29. (("sexual* transmi*" adj4 infection*) or "sexual* transmi* disease*" or STI or STIs or STD or STDs or STBBI or STBBIs or HIV or HepC or chlamydia or gonorrh?ea or syphili*).mp.
30. papillomaviridae/ or exp wart virus/
31. ("human papillomavirus" or HPV).mp.
32. sexual education/
33. ("sex* education" or "reproducti* education").mp.
34. gender based violence/
35. domestic violence/ or battered woman/ or exp partner violence/
36. physical abuse/
37. exp sexual assault/ or exp rape/ or sexual abuse/
38. sexual crime/
39. ("gender-based violence" or "dating violence" or "partner violence" or "gender-based abuse" or "dating abuse" or "partner abuse" or "consensual sex" or rape* or "safe sex" or "sex work*").mp.
40. right to health/ or health care access/
41. health service/
42. "planned parenthood".mp.
43. ((child or teen* or adolescen*) adj2 sex).mp.
44. exp sexuality/ or exp sexual orientation/
45. exp homosexuality/
46. (sexuality or gay or lesbian* or homosexual* or transgender or intersex* or "gender expression" or "gender identity" or two-spirit* or bisexual* or heterosexual* or LGBT or LGBTQ2 or "sexual orientation" or "trans health" or "trans rights").mp.
47. circumcision/
48. exp female genital mutilation/
49. (circumcis* or "genital mutilat*" or "genital cutting*").mp.
50. or/7-49
51. developing country/ or low income country/ or middle income country/
52. ("low* middle income countr*" or LMIC or LMICs or "low income countr*" or "middle income countr*").mp.

53. (AFGHANISTAN or afghan? or ALBANIA* or ALGERIA* or "AMERICAN SAMOA*" or ANGOLA* or ARGENTINA* or ARMENIA* or AZERBAIJAN or BANGLADESH* or BELARUS or BELIZE or BENIN or BHUTAN* or BOLIVIA* or "BOSNIA AND HERZEGOVINA" or BOTSWANA* or BRAZIL* or BULGARIA* or "BURKINA FASO" or BURUNDI or "CABO VERDE" or CAMBODIA* or CAMEROON or "CENTRAL AFRICAN REPUBLIC" or CHAD or CHINA or chinese or COLOMBIA* or COMOROS or CONGO* or "COSTA RICA*" or "COTE D'IVOIRE" or "ivory coast" or CUBA* or DJIBOUTI or DOMINICA* or "DOMINICAN REPUBLIC" or ECUADOR* or EGYPT* or "EL SALVADOR" or "EQUATORIAL GUINEA" or ERITREA* or ESWATINI or ETHIOPIA* or FIJI* or GABON or GAMBIA* or GEORGIA* or GHANA* or GRENADA* or GUATEMALA* or GUINEA or "GUINEA-BISSAU" or GUYANA* or HAITI* or HONDURAS or INDIA* or INDONESIA* or IRAN* or IRAQ* or JAMAICA* or JORDAN* or KAZAKHSTAN* or KENYA* or KIRIBATI or KOREA* or KOSOVO* or "KYRGYZ REPUBLIC" or LAO* or LEBANON or Lebanese or LESOTHO or LIBERIA* or LIBYA* or MADAGASCAR* or MALAWI* or MALAYSIA* or MALDIVES or MALI or "MARSHALL ISLANDS" or MAURITANIA* or MEXICO or mexican or MICRONESIA or MOLDOVA* or MONGOLIA* or MONTENEGRO or MOROCCO or moroccan* or MOZAMBIQUE or MYANMAR or NAMIBIA* or NEPAL* or NICARAGUA* or NIGER or NIGERIA* or "NORTH MACEDONIA" or PAKISTAN* or "PAPUA NEW GUINEA" or PARAGUAY or PERU* or PHILIPPINES or filipino* or RUSSIA* or RWANDA* or SAMOA* or "SAO TOME AND PRINCIPE" or SENEGAL* or SERBIA* or "SIERRA LEONE" or "SOLOMON ISLANDS" or SOMALIA* or "SOUTH AFRICA*" or "SRI LANKA*" or "ST. LUCIA*" or "ST. VINCENT AND THE GRENADINES" or SUDAN* or SURINAME or SYRIA* or TAJIKISTAN* or TANZANIA* or THAILAND or thai or "TIMOR-LESTE" or TOGO or TONGA* or TUNISIA* or TURKEY or turkish or TURKMENISTAN* or TUVALU or UGANDA* or UKRAINE or ukrainian* or UZBEKISTAN* or VANUATU or VENEZUELA* or VIETNAM or Vietnamese or "WEST BANK AND GAZA" or YEMEN or ZAMBIA* or ZIMBABWE*).mp.
54. 51 or 52 or 53
55. (intervention* or approach* or strateg* or program* or service* or plan* or policy or policies or scheme* or access* or procedure*).ti,ab.
56. 1 and 6 and 50 and 54 and 55

Number of results: 75

8. **Database:** PsycINFO via OVID (1806 - Present)
Date of Search: Feb 19 2021
APA PsycInfo <1806 to February Week 2 2021>

Line Number	Search terms	Number of Results
1	coronavirus/	2209

2	(coronavirus or "corona virus" or COVID19 or COVID-19 or COVID).mp.	3948
3	((novel or new or nouveau) adj2 (CoV on nCoV or covid or coronavirus* or "corona virus" or Pandemi*)).mp.	343
4	("SARS-Cov2" or "sars-cov-2").mp.	525
5	1 or 2 or 3 or 4	3980
6	Adolescent Mothers/ or Adolescent Development/ or Adolescent Characteristics/ or Adolescent Behavior/ or Adolescent Fathers/ or Adolescent Attitudes/ or Adolescent Health/	76660
7	(Adolescen* or teen* or youth? or "young people" or "younger people" or "young adult*" or "young wom?n" or "young m?n" or youngster* or "school age*" or student* or "middle schooler*" or "high schooler*" or "secondary school").mp.	1228863
8	6 or 7	1228863
9	sexual health/ or safe sex/	3958
10	exp psychosexual behavior/ or exp gender identity/ or exp human courtship/ or exp human mate selection/ or exp orgasm/ or exp sex/ or exp sexual arousal/ or exp sexual function disturbances/ or exp "sexual intercourse (human)"/ or exp sexual orientation/ or exp sexual attitudes/	238068
11	exp reproductive health/ or exp birth/ or exp birth control/ or exp family planning/ or exp fertility/ or exp infertility/ or exp menstruation/ or exp pregnancy/ or exp sexual reproduction/	65787
12	puberty/ or menarche/	3247
13	exp contraceptive devices/	5956
14	exp sexual development/	1783
15	gynecology/ or exp obstetrics/	3464
16	("Sexual health" or "sexual behavio?r*" or "sexual rights" or "sexual wellness" or "sexual wellbeing" or "sexual well-being").mp.	49990
17	pregnancy/ or adolescent pregnancy/	26106
18	((adolescen* or teen*) adj2 pregnan*).mp.	11060
19	induced abortion/	2701
20	("Reproductive health" or "reproductive justice" or "reproductive right*" or contracepti* or condom* or "birth control" or	177187

	IUD or "intrauterine device?" or "family planning" or abortion* or abstinen* or fertility or gynecolog* or obstetric* or OBGYN or menstruation or menarche or menstrual or puberty or "sexual* matur*" or maternal or maternity or prenatal or pre-natal or antenatal or postnatal or post-natal or postpartum or post-partum).mp.	
21	exp sexually transmitted diseases/ or exp hiv/ or exp syphilis/	47055
22	(("sexual* transmi*" adj4 infection*) or "sexual* transmi* disease*" or STI or STIs or STD or STDs or STBBI or STBBIs or HIV or HepC or chlamydia or gonorrh?ea or syphili*).mp.	61748
23	human papillomavirus/	1415
24	("human papillomavirus" or HPV or "genital wart*").mp.	2107
25	sex education/	3725
26	("sex* education" or "reproducti* education").mp.	8802
27	intimate partner violence/ or physical abuse/ or exp sexual abuse/	42472
28	rape/	5645
29	("gender-based violence" or "dating violence" or "partner violence" or "gender-based abuse" or "dating abuse" or "partner abuse" or "consensual sex" or rape* or "safe sex" or "sex work*").mp.	35628
30	((child or teen* or adolescen*) adj2 sex).mp.	3713
31	planned parenthood.mp.	173
32	sexuality/	16400
33	exp lgbtq/ or exp homosexuality/	31766
34	(sexuality or gay or lesbian* or homosexual* or transgender or intersex* or "gender expression" or "gender identity" or two-spirit* or bisexual* or heterosexual* or LGBT or LGBTQ2 or "sexual orientation" or "trans health" or "trans rights").mp.	102344
35	circumcision/	834
36	(circumcis* or "genital mutilat*" or "genital cutting*").mp.	1571
37	or/9-36	528308
38	developing countries/	5665

39	("low* middle income countr*" or LMIC or LMICs or "low income countr*" or "middle income countr*").mp.	5076
40	(AFGHANISTAN or afghan? or ALBANIA* or ALGERIA* or "AMERICAN SAMOA*" or ANGOLA* or ARGENTINA* or ARMENIA* or AZERBAIJAN or BANGLADESH* or BELARUS or BELIZE or BENIN or BHUTAN* or BOLIVIA* or "BOSNIA AND HERZEGOVINA" or BOTSWANA* or BRAZIL* or BULGARIA* or "BURKINA FASO" or BURUNDI or "CABO VERDE" or CAMBODIA* or CAMEROON or "CENTRAL AFRICAN REPUBLIC" or CHAD or CHINA or chinese or COLOMBIA* or COMOROS or CONGO* or "COSTA RICA*" or "COTE D'IVOIRE" or "ivory coast" or CUBA* or DJIBOUTI or DOMINICA* or "DOMINICAN REPUBLIC" or ECUADOR* or EGYPT* or "EL SALVADOR" or "EQUATORIAL GUINEA" or ERITREA* or ESWATINI or ETHIOPIA* or FIJI* or GABON or GAMBIA* or GEORGIA* or GHANA* or GRENADA* or GUATEMALA* or GUINEA or "GUINEA-BISSAU" or GUYANA* or HAITI* or HONDURAS or INDIA* or INDONESIA* or IRAN* or IRAQ* or JAMAICA* or JORDAN* or KAZAKHSTAN* or KENYA* or KIRIBATI or KOREA* or KOSOVO* or "KYRGYZ REPUBLIC" or LAO* or LEBANON or Lebanese or LESOTHO or LIBERIA* or LIBYA* or MADAGASCAR* or MALAWI* or MALAYSIA* or MALDIVES or MALI or "MARSHALL ISLANDS" or MAURITANIA* or MEXICO or mexican or MICRONESIA or MOLDOVA* or MONGOLIA* or MONTENEGRO or MOROCCO or moroccan* or MOZAMBIQUE or MYANMAR or NAMIBIA* or NEPAL* or NICARAGUA* or NIGER or NIGERIA* or "NORTH MACEDONIA" or PAKISTAN* or "PAPUA NEW GUINEA" or PARAGUAY or PERU*	327902

	or PHILIPPINES or filipino* or RUSSIA* or RWANDA* or SAMOA* or "SAO TOME AND PRINCIPE" or SENEGAL* or SERBIA* or "SIERRA LEONE" or "SOLOMON ISLANDS" or SOMALIA* or "SOUTH AFRICA*" or "SRI LANKA*" or "ST. LUCIA*" or "ST. VINCENT AND THE GRENADINES" or SUDAN* or SURINAME or SYRIA* or TAJIKISTAN* or TANZANIA* or THAILAND or thai or "TIMOR-LESTE" or TOGO or TONGA* or TUNISIA* or TURKEY or turkish or TURKMENISTAN* or TUVALU or UGANDA* or UKRAINE or ukrainian* or UZBEKISTAN* or VANUATU or VENEZUELA* or VIETNAM or Vietnamese or "WEST BANK AND GAZA" or YEMEN or ZAMBIA* or ZIMBABWE*).mp.	
41	38 or 39 or 40	332446
42	5 and 8 and 37 and 41	7
